# Creatine and essential amino acids with exercise in obesity: an immunometabolic perspective on the muscle–adipose–brain axis

**DOI:** 10.3389/fimmu.2026.1931736

**Published:** 2026-10-19

**Authors:** Yongjun Zhao, Juan Zhao, Congcong Xie, Xuxia Yang, Jianying Li

**Affiliations:** 1 Institute of Sports Science, Shanxi University, Taiyuan, Shanxi, China; 2 Department of Physical Education and Health, Lyuliang University, Lyuliang, Shanxi, China; 3 Rehabilitation Department, Taiyuan Peace Hospital, Taiyuan, Shanxi, China; 4 Physical Education School of Shaanxi Normal University, Xi’an, Shaanxi, China; 5 School of Physical Education, North University of China, Taiyuan, Shanxi, China

**Keywords:** creatine, essential amino acid, exercise, immunometabolism, neurobehavioral adaptations, overweight

## Abstract

Obesity is associated with anabolic resistance, skeletal muscle dysfunction, adipose tissue dysregulation, chronic low-grade inflammation, and altered brain metabolism. Exercise is a primary strategy for counteracting these disturbances, while nutritional interventions may complement exercise-induced adaptations. Creatine and essential amino acids (EAAs) have established roles in muscle bioenergetics and protein synthesis, respectively, but their combined use with exercise and potential effects on the interconnected muscle–adipose–brain axis in obesity remain incompletely characterized. This review examines mechanistic and clinical evidence for creatine and EAA supplementation alongside exercise, with emphasis on muscle anabolism, body composition, adipose remodeling, immunometabolism, and muscle–adipose–brain communication. Exercise provides the principal physiological stimulus for muscle adaptation, whereas EAAs, particularly leucine, stimulate muscle protein synthesis through amino acid-sensing and mTORC1-related pathways. Creatine increases phosphocreatine availability and may enhance training capacity, strength, and lean mass when combined with resistance exercise. Available evidence suggests potentially complementary, rather than established synergistic, effects of creatine and EAAs with exercise on muscle preservation, functional capacity, and body composition. Beyond muscle, exercise-derived myokines, exerkines, adipokines, and metabolites provide plausible mechanisms linking peripheral adaptations to adipose tissue and the brain. Creatine may support cellular and cerebral bioenergetics, whereas EAAs influence amino acid availability and protein turnover. However, direct evidence that these interventions modify obesity-associated neuroinflammation, depression, or cognitive dysfunction remains limited. Overall, current evidence supports exercise as the central intervention, with creatine and EAAs representing potentially complementary nutritional strategies. Evidence for integrated effects on adipose–brain communication, immunometabolism, and neurobehavioral outcomes remains preliminary and is derived substantially from indirect or preclinical findings. Future randomized trials should directly compare individual and combined interventions, establish optimal dosing and timing, and integrate mechanistic biomarkers with clinically relevant metabolic and neurobehavioral outcomes.

## 1 Introduction

Obesity is a chronic metabolic disorder marked by excessive accumulation of adipose tissue, reduced metabolic flexibility, insulin resistance, persistent low-grade inflammation, and steadily worsening impairments in tissue function. Notably, obesity is also linked to defective skeletal muscle protein turnover and diminished anabolic responsiveness, factors that may contribute to reductions in muscle quality and functional capacity (1). These alterations can be conceptualized as part of an integrated muscle–adipose–brain network, in which metabolic substrates, myokines, adipokines, exerkines, metabolites, and immune mediators all contribute to signaling between peripheral tissues and the brain (2, 3). Therefore, disruption of this inter-organ communication may contribute concurrently to impaired muscle function, adipose tissue dysfunction, systemic inflammation, and altered brain metabolism.

Skeletal muscle is a primary site for glucose uptake as well as energy expenditure, and it also acts as an endocrine organ that can affect remote tissues via myokines and exercise-responsive metabolites. In obesity, insulin resistance, lipid accumulation within muscle, mitochondrial impairment, and persistent inflammation may compromise muscle metabolic flexibility and weaken adaptive responses to nutritional and contractile cues (4, 5). Accordingly, exercise continues to be a central strategy for enhancing muscle metabolic health, physical function, and body composition. Myokines and other exerkines induced by exercise can likewise modulate adipose tissue, immune function, and potentially brain physiology, offering a biological framework for inter-organ communication during physical activity (2, 3). Even so, the magnitude of these adaptations differs across individuals, especially when obesity coexists with profound metabolic derangements, reduced physical capacity, or pronounced anabolic resistance. This heterogeneity has therefore increased interest in nutritional approaches designed to support, rather than substitute for, exercise-driven adaptation.

Creatine and essential amino acids (EAAs) are two nutritional strategies with distinct mechanisms of action in skeletal muscle. Creatine supplementation raises the availability of intramuscular phosphocreatine and may help rapidly regenerate adenosine triphosphate (ATP) during repeated high-intensity muscle contractions, which can improve resistance-exercise performance and contribute to training-related gains in strength and fat-free mass (6). EAAs, especially leucine, act as both substrates and nutrient signals that promote muscle protein synthesis via amino acid–responsive signaling pathways involving the mTORC1 complex (7, 8). These mechanisms indicate possible complementarity: exercise acts as the main physiological trigger for adaptation, EAAs help maintain amino acid availability and anabolic signaling, while creatine contributes to cellular energy buffering and the ability to carry out repeated high-intensity contractions. However, direct evidence showing genuine synergy between creatine and EAAs, especially when they are combined with exercise in obesity, remains scarce. In addition, effects that extend beyond skeletal muscle, including adipose tissue remodeling, immunometabolic regulation, neuroinflammation, and neurobehavioral outcomes, are supported by heterogeneous evidence from human, animal, and mechanistic studies and should therefore be interpreted according to the level of evidence.

The central purpose of this review is therefore not to reassess creatine, EAA nutrition, or exercise in isolation, but to evaluate how their interplay may affect the coordinated muscle–adipose–brain axis in obesity. We separate well-established findings in humans from largely preclinical results and distinguish mechanisms supported by experimental data from those that are emerging or still hypothetical. Emphasis is placed on (i) obesity-associated impairment of communication within the muscle–adipose–brain axis; (ii) exercise as the main physiological factor that modifies this network; (iii) the complementary contributions of creatine and EAAs to supporting muscle adaptation; (iv) circulating metabolites and immune-cell reprogramming as possible mediators of communication between organs; and (v) potential consequences for neuroinflammation, depression, cognition, and the management of body composition. By bringing these elements together, this review seeks to clarify what is currently supported by evidence, determine where the evidence remains indirect or limited to preclinical work, and set priorities for future clinical research.

## 2 Obesity as a disorder of muscle–adipose–brain crosstalk

### 2.1 Obesity, chronic inflammation and immunometabolism

Obesity is a chronic immunometabolic state in which adipose expansion, immune-cell remodeling, and metabolic dysfunction reinforce one another (9, 10). Persistent low-grade inflammation contributes to insulin resistance, impaired tissue adaptation, and systemic metabolic dysfunction.

As adipocytes expand and adipose tissue becomes metabolically stressed, local hypoxia, endoplasmic-reticulum stress, oxidative stress, and extracellular-matrix remodeling promote immune-cell recruitment. Macrophage accumulation around dysfunctional or dying adipocytes and changes in regulatory immune populations contribute to a sustained inflammatory phenotype (10, 11). The field of immunometabolism provides a framework for understanding the reciprocal relationship between immune activation and cellular metabolism. In obesity, nutrient excess can engage inflammatory pathways involving nuclear factor-κB, c-Jun N-terminal kinase, and the NLRP3 inflammasome, thereby promoting cytokine production and impairing metabolic signaling (12). These alterations extend beyond adipose tissue to skeletal muscle, liver, pancreatic islets, and the central nervous system, supporting the view of obesity as a systemic immunometabolic disorder. Importantly, immune activation is accompanied by changes in cellular substrate utilization, mitochondrial function, and redox balance. Such metabolic reprogramming can sustain inflammatory signaling and further impair tissue metabolic flexibility. This reciprocal relationship between immune metabolism and tissue metabolism provides an important mechanistic link between obesity and impaired adaptation to nutritional and exercise stimuli.

### 2.2 Muscle–adipose endocrine communication

Skeletal muscle and adipose tissue function as endocrine organs that communicate bidirectionally through myokines, adipokines, metabolites, and other circulating mediators (2). Under physiological conditions, this communication contributes to coordinated regulation of glucose and lipid metabolism, tissue remodeling, and inflammatory tone. In obesity, these signals become altered and may contribute to reciprocal dysfunction of muscle and adipose tissue. Healthy skeletal muscle secretes myokines that can influence adipocyte metabolism, mitochondrial function, lipid handling, and inflammatory responses. Conversely, adipose-derived signals regulate skeletal-muscle insulin sensitivity, substrate metabolism, and protein turnover (13, 14). Adiponectin generally promotes fatty-acid oxidation and insulin sensitivity, whereas altered leptin, resistin, and other adipose-derived inflammatory signals in obesity may contribute to metabolic dysfunction and impaired muscle adaptation (13, 14). As obesity advances, adipose-derived inflammatory and lipotoxic signals can impair muscle metabolism, while metabolic dysfunction in skeletal muscle may alter its capacity to support healthy adipose remodeling (2). Thus, obesity may transform muscle–adipose communication from a coordinated metabolic network into a maladaptive feedback loop.

### 2.3 Exercise-induced myokines, adipokines and exerkines

Exercise provokes systemic responses that extend beyond energy expenditure. Contracting skeletal muscle and other tissues release myokines, adipokines, metabolites, extracellular vesicles, and other exercise-responsive mediators, collectively contributing to the broader exerkine network (15, 16). These signals provide a potential mechanism through which exercise communicates with tissues beyond skeletal muscle. Among the tissues responsive to exercise, skeletal muscle emerges as the primary endocrine organ, releasing exercise-responsive myokines whose secretion depends on exercise mode, intensity, duration, training status, and nutritional context (2, 15). These factors can influence glucose and lipid metabolism, mitochondrial adaptation, immune signaling, and tissue remodeling. Additionally, adipose tissue responds to exercise through changes in adipokine secretion, lipid mobilization, and tissue remodeling, while circulating metabolites and extracellular vesicles may provide additional routes for inter-organ communication (3). The magnitude and biological consequences of these responses may differ according to exercise characteristics and metabolic status, which is particularly relevant in obesity. Accordingly, exercise provides a physiological context in which muscle–adipose communication can be remodeled; its potential interaction with nutritional strategies is considered in subsequent sections.

### 2.4 Muscle–adipose–brain axis

The muscle–adipose–brain axis provides a framework for understanding bidirectional communication between peripheral metabolic tissues and the central nervous system through endocrine, neural, immune, and metabolic signals (17). Skeletal muscle and adipose tissue convey information about nutrient availability, energy status, inflammatory tone, and tissue activity, whereas the brain integrates these signals and regulates appetite, energy expenditure, glucose homeostasis, autonomic activity, and behavioral responses through hypothalamic, brainstem, and autonomic circuits (18). Myokines, adipokines, metabolites, lipid mediators, extracellular vesicles, and neuroendocrine signals therefore contribute to a dynamic network rather than isolated muscle-to-brain or adipose-to-brain pathways (19). In obesity, chronic nutrient excess, inflammation, insulin and leptin resistance, mitochondrial dysfunction, and altered peripheral secretory profiles can disturb this communication. These abnormalities may simultaneously impair skeletal-muscle and adipose-tissue metabolism while altering central regulation of energy homeostasis, providing a conceptual basis for considering obesity as a disorder of inter-organ communication.

### 2.5 Neuroinflammation and mitochondrial dysfunction in obesity

Obesity is associated not only with peripheral metabolic dysfunction but also with alterations in central metabolic and neuroimmune homeostasis. Chronic nutrient excess, circulating inflammatory mediators, and metabolic stress can affect microglia, astrocytes, and neurons, particularly within hypothalamic regions involved in energy regulation (20). Persistent glial activation may increase inflammatory signaling and interfere with neuronal insulin and leptin pathways, thereby contributing to impaired regulation of appetite and energy expenditure (21, 22). Mitochondrial dysfunction may further connect metabolic stress to neuroinflammation. Excess nutrient availability can increase mitochondrial oxidative stress, alter electron-transport activity and mitochondrial dynamics, and impair cellular ATP production (23). Because neuronal function depends heavily on tightly regulated energy metabolism, impaired mitochondrial homeostasis may compromise synaptic function and increase susceptibility to inflammatory and oxidative stress. These observations provide a mechanistic link between obesity-associated peripheral metabolic dysfunction and central nervous-system abnormalities. Importantly, these pathways should not be interpreted as evidence that creatine or EAA supplementation directly reverses obesity-associated neuroinflammation or brain dysfunction. Rather, they define biological processes that may be influenced indirectly by exercise and nutritional interventions and therefore provide the mechanistic background for the intervention-focused sections that follow.

## 3 Mechanisms of muscle anabolism in obesity

### 3.1 Skeletal muscle protein synthesis and anabolic resistance

Skeletal muscle protein synthesis (MPS) is a major determinant of muscle maintenance and adaptation. In people with overweight or obesity, the response of muscle protein metabolism to nutritional and exercise stimuli can be attenuated, a phenomenon commonly referred to as anabolic resistance (24–26). This impaired responsiveness may involve reduced activation of translational signaling after amino acid ingestion or resistance exercise and can contribute to impaired muscle quality and functional adaptation (**Figure 1**). Several obesity-associated abnormalities may contribute to anabolic resistance, including chronic low-grade inflammation, insulin resistance, intramuscular lipid accumulation, mitochondrial dysfunction, and altered nutrient sensing. Elevated concentrations of free fatty acids and inflammatory mediators can interfere with insulin and amino acid signaling and may impair the coordinated activation of pathways involved in muscle protein turnover (24–26). These alterations are particularly relevant during weight-loss interventions, when reduced energy intake may increase the challenge of preserving lean tissue. Thus, the attenuated anabolic response associated with obesity provides an important biological rationale for interventions that combine an adequate protein/EAA supply with resistance exercise. The potential contribution of creatine is considered separately below because its primary role involves cellular energy buffering and exercise capacity rather than direct provision of amino acid substrate.

**Figure 1:**
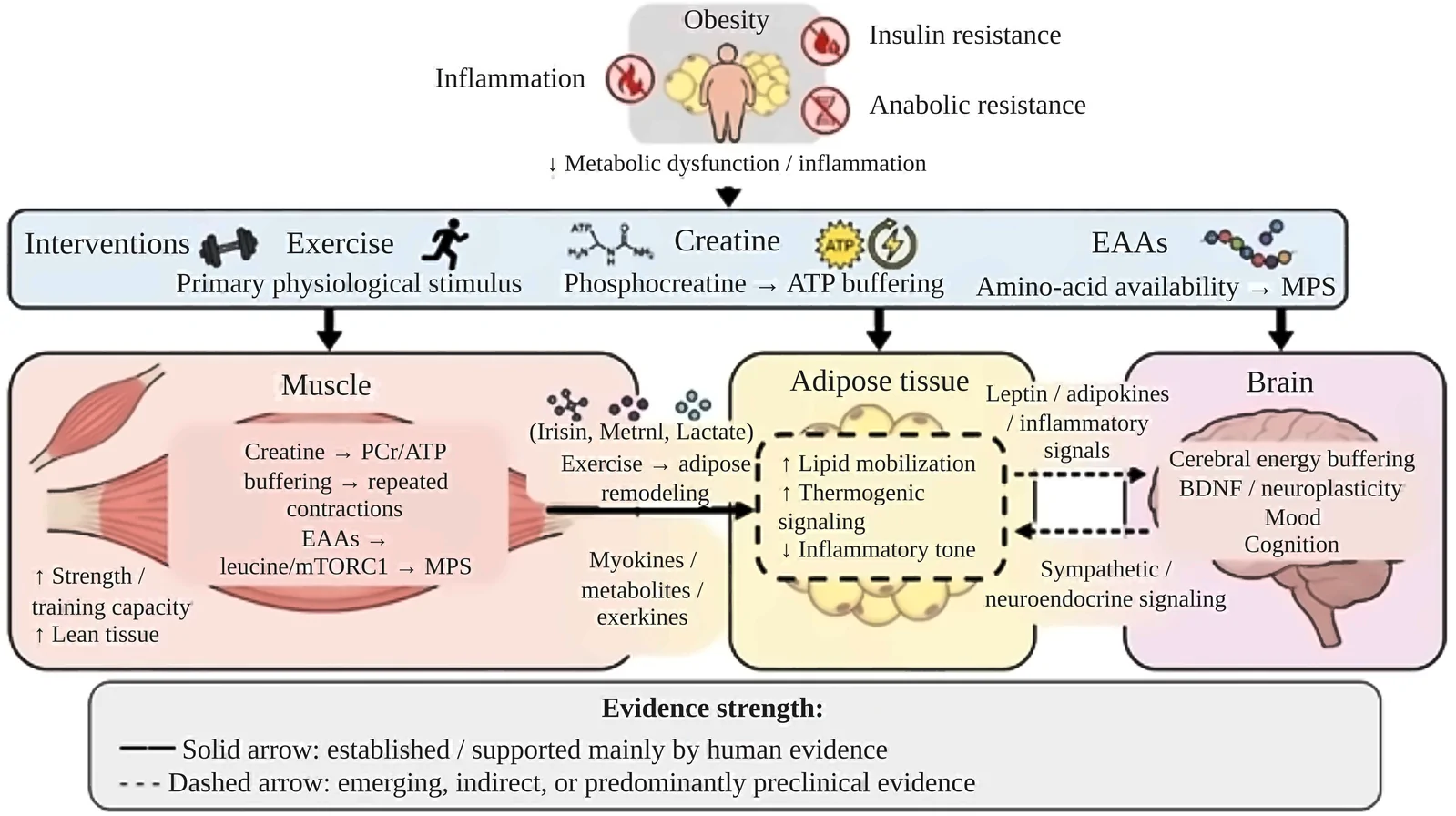
Exercise-centered regulation of the muscle–adipose–brain axis by creatine and essential amino acids in obesity. Obesity is associated with anabolic resistance, insulin resistance, chronic low-grade inflammation, and impaired communication among skeletal muscle, adipose tissue, and the brain. Exercise acts as the primary physiological stimulus for remodeling this network. Creatine mainly supports skeletal muscle bioenergetics by increasing phosphocreatine availability and facilitating ATP regeneration during repeated contractions, whereas essential amino acids (EAAs), particularly leucine, provide substrates and nutrient signals that promote muscle protein synthesis through mTORC1-related pathways. Exercise-induced myokines, exerkines, and metabolites may subsequently influence adipose tissue remodeling, inflammatory signaling, and brain function. Creatine may also support cellular and cerebral energy buffering, while EAAs may affect nutrient-sensitive signaling and amino acid availability in the brain. Solid arrows indicate mechanisms supported by relatively stronger human evidence; dashed arrows represent emerging, indirect, or predominantly preclinical mechanisms. The image emphasizes potentially complementary actions of exercise, creatine, and EAAs rather than an established synergistic effect across the muscle-adipose-brain axis.

### 3.2 Nutrient- and exercise-responsive anabolic signaling

The IGF-1–PI3K–Akt–mTORC1 network integrates hormonal, nutritional, and mechanical signals that regulate muscle protein synthesis and remodeling (27, 28). In metabolic dysfunction, impaired insulin and IGF-1 signaling can reduce Akt activation and downstream mTORC1 signaling, potentially limiting the phosphorylation of translational regulators such as p70S6 kinase and 4E-BP1. Amino acid availability, particularly leucine availability, provides an additional nutrient-sensitive input into mTORC1 signaling, whereas mechanical loading activates overlapping but not identical anabolic pathways. Consequently, the anabolic response to exercise depends on the interaction between mechanical stimuli, nutrient availability, and the metabolic state of the muscle. Obesity-associated insulin resistance and altered nutrient sensing may therefore reduce the efficiency with which these signals are translated into muscle protein accretion (27, 28). This signaling framework is relevant to the present review because it provides a mechanistic basis for examining how exercise, EAA availability, and cellular energy status may converge on muscle adaptation. However, the extent to which creatine and EAAs jointly modify these pathways in obesity remains insufficiently established and should not be inferred from mechanistic studies alone.

### 3.3 Lipotoxicity, mitochondrial dysfunction and impaired muscle adaptation

Obesity-associated lipid oversupply can increase intramyocellular lipid accumulation and alter mitochondrial and insulin signaling, thereby creating a metabolic environment that is less favorable for muscle adaptation (4, 29, 30). Lipotoxic intermediates such as ceramides and diacylglycerols have been implicated in impaired insulin signaling, while oxidative stress and chronic inflammation may further compromise mitochondrial function and muscle remodeling. These alterations may also affect the capacity of skeletal muscle to respond to repeated contractile stimuli by disrupting energy metabolism, protein turnover, and tissue repair. Although exercise remains a potent stimulus for maintaining muscle function, the magnitude of adaptation may vary according to the severity of metabolic dysfunction, baseline physical capacity, and nutritional status. These mechanisms establish the metabolic context in which nutritional support may complement exercise. The specific evidence for creatine and EAA supplementation, their interaction with exercise, and their potential effects beyond skeletal muscle are addressed in the following sections.

## 4 Creatine supplementation and exercise

### 4.1 Human evidence for creatine with exercise in overweight and obesity

Creatine supplementation, particularly when combined with resistance exercise, has been investigated as a strategy to support increases in fat-free mass, strength, and physical function. Resistance exercise itself remains the primary stimulus for muscle adaptation, whereas creatine supplementation increases intramuscular phosphocreatine availability and may facilitate repeated high-intensity contractions (6, 31). Human studies in people with overweight or obesity generally suggest that creatine can augment some exercise-induced improvements in strength and lean tissue, although the magnitude of benefit varies according to training status, intervention duration, baseline diet, and clinical phenotype. Importantly, these findings should not be interpreted as evidence that creatine independently reverses obesity-associated anabolic resistance or produces clinically meaningful fat loss. The principal evidence supports its use as an adjunct to structured exercise, particularly resistance training, for preserving or increasing lean tissue and improving strength-related outcomes (6, 31). The effects on body fat are less consistent and appear to depend strongly on the accompanying exercise and dietary intervention. Accordingly, creatine should primarily be considered in this context as a nutritional strategy that may enhance the capacity to train and support muscle adaptation, rather than as a direct anti-obesity treatment.

### 4.2 Creatine, muscle bioenergetics and adaptation

Creatine is integral to skeletal-muscle energy metabolism because the phosphocreatine system provides a rapidly available reservoir for ATP regeneration during high-intensity contractions. Supplementation increases intramuscular creatine and phosphocreatine stores, thereby supporting force production, repeated exercise bouts, and training capacity (6, 31). These bioenergetic effects provide a plausible explanation for the greater improvements in strength and fat-free mass observed in some creatine-plus-exercise interventions. Beyond ATP buffering, creatine has been proposed to influence cellular processes involved in muscle adaptation, including cellular hydration, satellite-cell activity, muscle protein turnover, and exercise-related signaling. However, these mechanisms should be distinguished from the more firmly established effect of creatine on intramuscular creatine/phosphocreatine availability. Evidence that creatine directly activates canonical anabolic pathways in humans with obesity remains less consistent than evidence for its effects on exercise performance and strength. This distinction is particularly relevant to the present review. Exercise provides the mechanical stimulus for muscle remodeling, whereas creatine primarily supports the energetic demands of repeated contraction. Thus, the biological rationale for their combination is based on potentially complementary functions rather than on a demonstrated molecular synergy.

### 4.3 Creatine beyond skeletal muscle: adipose and hepatic evidence

Evidence for effects of creatine beyond skeletal muscle is substantially more dependent on preclinical models and should therefore be interpreted separately from human exercise evidence. In mice fed a high-fat diet, creatine supplementation has been reported to improve several metabolic parameters, including insulin sensitivity and glucose homeostasis, while increasing markers of brown-adipose thermogenesis such as UCP1 and CIDEA. Creatine was also associated with changes in lipid handling and lipophagy in metabolic tissues (32). These findings suggest that creatine metabolism may participate in the regulation of adipose energy expenditure, but they do not establish equivalent effects in people with obesity. Consistent with this possibility, adipose-specific deletion of glycine amidinotransferase (GATM), a key enzyme in creatine biosynthesis, increased susceptibility to diet-induced obesity and impaired adaptive thermogenesis in mice. Dietary creatine restored metabolic rate and thermogenic responsiveness in this model (33). This genetic evidence provides mechanistic support for a role of adipose creatine metabolism in energy expenditure, although translation to human obesity remains uncertain. Additional rodent studies have reported effects of creatine supplementation on high-fat-diet-induced hepatic steatosis. In one model, creatine reduced hepatic lipid accumulation and lipid peroxidation while restoring hepatic S-adenosylmethionine concentrations and the expression of PPARα and β-oxidation-related genes (34). In another, five weeks of creatine supplementation increased lipoprotein secretion and mitochondrial function and attenuated hepatic lipid accumulation (35). These observations broaden the potential metabolic actions of creatine beyond skeletal muscle, but they remain predominantly preclinical and should not be presented as established clinical effects in obesity. A separate high-fat/high-fructose rat study further illustrates the importance of this distinction. Creatine supplementation reduced IL-1β and IL-6 concentrations but did not prevent soleus muscle atrophy or the increased expression of MuRF1 and atrogin-1, despite the presence of obesity-associated inflammation and muscle catabolism (36). Thus, anti-inflammatory effects observed in selected animal models do not necessarily translate into preservation of muscle mass or prevention of obesity-associated muscle dysfunction.

### 4.4 Creatine and brain bioenergetics: relevance to neurobehavioral outcomes

Creatine is also involved in brain energy metabolism, where the creatine–phosphocreatine system contributes to ATP buffering in neurons and other brain cells. Experimental studies indicate that altered creatine availability can influence mitochondrial function, oxidative stress, and cellular resilience, providing a biological rationale for investigating creatine in conditions characterized by impaired cerebral bioenergetics (37–39). Human studies have additionally examined creatine as an adjunctive intervention for depression. Some clinical trials have reported improved depressive symptoms when creatine was added to antidepressant treatment, including studies in women receiving selective serotonin reuptake inhibitors, while neuroimaging studies have linked changes in cerebral phosphocreatine with depressive symptom severity (40, 41). However, these findings derive from selected clinical populations and do not demonstrate that creatine treats obesity-associated depression specifically. Creatine has also been investigated for cognitive performance under conditions of elevated energetic demand, including sleep deprivation, hypoxia, and substantial cognitive load (42, 43). Although these findings support a possible role for cerebral bioenergetics, direct evidence that creatine supplementation improves cognition or neuroinflammation in people with obesity remains limited. Therefore, the potential brain effects of creatine should currently be regarded as an emerging area of investigation rather than an established component of obesity management. Taken together, human evidence most consistently supports creatine as an adjunct to exercise for muscle-related outcomes, whereas effects on adipose tissue, liver, inflammation, and brain function are supported to varying degrees by preclinical or indirect clinical evidence. This evidence hierarchy is important when considering how creatine may contribute to the broader muscle–adipose–brain framework developed later in the review.

## 5 EAA supplementation and exercise

### 5.1 Effects of EAA supplementation on muscle mass, strength, and functional performance in overweight and obesity

EAA supplementation may support muscle preservation and functional adaptation in overweight and obesity, particularly when adequate amino acid availability is combined with an appropriate exercise stimulus. However, evidence for additive or synergistic effects of EAA supplementation with exercise remains limited and heterogeneous. EAAs provide essential substrates for MPS, while leucine also functions as a nutrient signal that activates anabolic pathways. This mechanism is potentially relevant to obesity, in which anabolic resistance can attenuate the response of skeletal muscle to nutritional and contractile stimuli. Human studies provide some evidence that EAA-containing interventions can improve body composition or functional outcomes, although the available trials differ substantially in population, formulation, intervention duration, and concomitant lifestyle treatment. A double-blind randomized controlled trial investigated an EAA-fortified meal replacement (EMR) in older adults with overweight or obesity. Over four weeks, participants received either EMR or a standardized commercial meal replacement (Optifast^®^). Compared with Optifast^®^, EMR produced greater reductions in total body fat, visceral adipose tissue, and intrahepatic lipid, while thigh skeletal muscle cross-sectional area increased with EMR but decreased in the comparator group. The EMR group also showed a greater improvement in 6-minute walk distance, suggesting that an EAA-enriched nutritional intervention may help preserve muscle morphology and physical function during short-term weight reduction (44). Because the intervention involved a fortified meal replacement rather than isolated EAAs and was not necessarily combined with a standardized exercise program, these findings should not be interpreted as evidence of an EAA–exercise interaction.

Another investigation examined EAA supplementation in adolescents with polycystic ovary syndrome (PCOS) and hepatic steatosis. Twenty-one participants completed two 28-day intervention phases involving EAA supplementation or placebo. EAA supplementation did not significantly alter body mass but was associated with reductions in hepatic steatosis, plasma aspartate aminotransferase, triacylglycerol, and very-low-density lipoprotein–triacylglycerol. In contrast, indices of insulin sensitivity and VLDL-triacylglycerol derived from *de novo* lipogenesis were not significantly changed. Because dietary carbohydrate and total sugar intake also decreased during the EAA phase, attribution of these metabolic effects exclusively to EAA supplementation requires caution (45). Additional evidence comes from studies of older adults with sarcopenic obesity. In a randomized study of 48 men aged >60 years, participants received EAA supplementation alone or EAA supplementation combined with electroacupuncture. Both groups reduced body-fat percentage, whereas appendicular skeletal muscle index improved earlier and to a greater extent in the combined electroacupuncture–EAA group. These findings support the possibility that EAA availability may complement an anabolic stimulus, but the study does not establish a specific interaction between EAA supplementation and exercise because electroacupuncture was the additional intervention (46). A study of 68 women with grade I obesity evaluated 8 g/day of EAA supplementation during the re-educational phase following a very-low-energy ketogenic therapy. After fruit reintroduction, the EAA-supplemented group showed greater reductions in body weight, waist circumference, and fat mass and greater improvements in high-sensitivity C-reactive protein, muscle strength, phase angle, body cell mass, and relative muscle mass (47). These findings are relevant to muscle preservation during weight loss, although the multistage dietary intervention and absence of a standardized exercise component limit conclusions regarding an independent EAA effect or an EAA–exercise interaction.

Preclinical studies provide additional evidence for metabolic effects of EAA manipulation. In obese male Zucker rats, insect meal-based diets, with or without additional methionine, cysteine, or EAAs, reduced hepatic triacylglycerol and cholesterol compared with casein. Addition of individual sulfur-containing amino acids or EAAs did not substantially reverse these lipid-lowering effects, indicating that the observed hepatic response could not be attributed simply to the amino acid composition of the diet (48). This study therefore provides mechanistic context rather than direct evidence for EAA supplementation as a treatment for obesity. Similarly, in a chronic murine model, an EAA-enriched high-fat diet prevented or reversed several obesity-associated abnormalities, including excess adiposity, impaired glucose tolerance, mesenteric fat accumulation, intestinal barrier disruption, and inflammatory responses. EAA exposure was also associated with restoration of intestinal mitochondrial biogenesis and oxidative metabolism, accompanied by increased PGC-1α and electron-transport-chain gene expression. Complementary experiments in Caco-2 cells showed improved barrier function, mitochondrial respiratory capacity, antioxidant defenses, and reduced IL-8 secretion (49). Although these findings suggest that amino acid availability can influence intestinal immunometabolism, they remain predominantly preclinical and do not demonstrate that isolated EAA supplementation produces comparable effects in humans with obesity.

Conversely, a small clinical investigation of six patients with severe obesity who received oral EAA supplementation after jejunoileal bypass surgery found a modest improvement in markers of protein nutritional status but no prevention of progressive hepatic morphological abnormalities or liver dysfunction (50). This negative finding is important because it illustrates that EAA supplementation does not uniformly correct obesity-associated metabolic or hepatic abnormalities. Taken together, human evidence suggests that EAA-containing interventions may be useful for maintaining muscle-related outcomes during energy restriction or weight loss, with some studies also reporting favorable changes in adiposity and metabolic markers. Nevertheless, the evidence is heterogeneous, and direct trials comparing exercise alone with exercise plus isolated EAA supplementation in obesity remain insufficient.

### 5.2 Mechanisms of EAA-mediated muscle adaptation

The principal rationale for EAA supplementation in obesity is its capacity to increase the availability of essential amino acids required for muscle protein accretion and to activate nutrient-sensitive anabolic signaling. EAAs, particularly leucine, stimulate mTORC1-related signaling and translation initiation, thereby promoting MPS when sufficient amino acid availability is accompanied by an appropriate physiological stimulus. This mechanism is particularly relevant in obesity-associated anabolic resistance, in which insulin resistance, chronic inflammation, intramuscular lipid accumulation, and impaired nutrient sensing may reduce the efficiency of the muscle anabolic response. Mechanical loading provided by resistance exercise is an important complementary stimulus. Exercise activates mechanosensitive and intracellular signaling pathways, whereas EAAs provide substrates and nutrient signals that support subsequent protein synthesis. Thus, the two interventions may converge on muscle remodeling through partially distinct mechanisms. Evidence that EAA supplementation directly enhances skeletal-muscle mitochondrial ATP production in overweight or obese humans is considerably less established and should therefore be distinguished from mechanistic or preclinical observations (51). Adequate EAA availability may also favor a positive net protein balance by supporting MPS during periods in which protein breakdown is elevated, including energy restriction and recovery from exercise (52). However, whether this translates into superior long-term strength or functional adaptation when EAAs are added to a well-controlled exercise program in obesity remains incompletely established. Accordingly, current evidence supports a model of complementary biological actions rather than demonstrated molecular synergy: exercise provides the primary contractile stimulus, whereas EAAs support amino acid availability and nutrient-sensitive anabolic signaling.

### 5.3 EAAs, neurotransmitter synthesis, and brain metabolism

EAAs are essential for skeletal muscle protein synthesis, but their physiological effects extend to the central nervous system through amino acid transport, neurotransmitter precursor availability, nitrogen metabolism, and nutrient-sensitive signaling. Because the brain has limited capacity to synthesize several essential amino acids *de novo*, circulating amino acid availability and transport across the blood–brain barrier can influence central amino acid homeostasis (53, 54). Nevertheless, the relevance of these mechanisms to obesity-related neuroinflammation, depression, or cognitive dysfunction remains substantially less established than the role of EAAs in skeletal muscle. Among the EAAs, tryptophan is a precursor for serotonin synthesis. Its transport across the blood–brain barrier depends partly on its plasma concentration relative to competing large neutral amino acids (LNAAs), including leucine, isoleucine, valine, phenylalanine, and tyrosine, which share the LAT1 transport system. Consequently, changes in the circulating tryptophan-to-LNAA ratio can alter the availability of tryptophan for cerebral serotonin synthesis (53). Human nutritional studies provide proof of principle that manipulating dietary protein composition can modify this ratio. For example, tryptophan-rich α-lactalbumin has been reported to increase the plasma tryptophan-to-LNAA ratio and improve selected cognitive responses under conditions of psychological stress (55). However, these findings cannot be directly extrapolated to isolated EAA supplementation in obesity or interpreted as evidence of an antidepressant effect.

EAAs may also influence brain metabolism through mechanisms independent of neurotransmitter precursor availability. BCAAs participate in cerebral nitrogen metabolism, while leucine can function as a nutrient signal capable of engaging mTORC1-related pathways involved in neuronal protein synthesis and cellular plasticity (56). These mechanisms provide a plausible link between systemic amino acid availability and neuronal adaptation, but the physiological magnitude and clinical relevance of these effects in obesity remain uncertain. Obesity itself is associated with altered amino acid metabolism, insulin resistance, chronic inflammation, and changes in central nutrient sensing and blood–brain barrier function. These abnormalities could theoretically modify the availability or utilization of amino acids within the brain. However, whether restoring EAA availability can reverse these obesity-associated disturbances has not been adequately established in humans. Accordingly, the potential contribution of EAAs to brain health should currently be regarded as mechanistically plausible but clinically under-investigated. Overall, the strongest evidence for EAA supplementation in obesity concerns preservation of muscle-related outcomes during nutritional restriction and support of anabolic signaling. Evidence for effects on adipose tissue, intestinal or systemic immunometabolism, and particularly brain function is more heterogeneous and frequently derives from indirect or preclinical studies. Importantly, direct evidence that EAA supplementation modifies the muscle–adipose–brain axis in obesity is limited. These limitations provide the rationale for examining EAA supplementation together with exercise and creatine in the subsequent section, where complementary mechanisms and the current evidence hierarchy can be considered without assuming established synergy.

## 6 Creatine and EAA supplementation with exercise in obesity

### 6.1 Creatine, exercise, and muscle/body composition outcomes

The evidence reviewed in Section 4 indicates that creatine may enhance exercise-induced improvements in muscle strength, training capacity, and fat-free mass, but the magnitude of these effects varies across individuals with overweight or obesity. Rather than acting as an independent anabolic stimulus, creatine is more appropriately viewed as an energetic support that may enable a greater or more sustained training stimulus. Its potential value may therefore depend on the metabolic and nutritional context in which exercise is performed. Obesity-associated insulin resistance, chronic inflammation, altered nutrient sensing, and impaired muscle metabolic flexibility may reduce the responsiveness of skeletal muscle to both nutritional and contractile stimuli (57) (**Table 1**). Individuals with severe obesity or sarcopenic obesity may therefore require particular attention to the preservation of lean tissue during weight loss. Importantly, however, impaired responsiveness should not be interpreted as evidence that creatine is ineffective; rather, it may contribute to heterogeneity in the magnitude of adaptation. Dietary context may further influence the response to creatine. Experimental evidence suggests that high-fat feeding can alter anabolic signaling despite creatine-associated changes in IGF-1 and Akt-related pathways (58). Although these findings provide a mechanistic basis for considering metabolic phenotype and diet quality, their translation to humans with obesity remains uncertain. Another explanation for inconsistent clinical findings is the timing and physiological context of the intervention. Studies conducted during the early postoperative phase following bariatric surgery failed to demonstrate additional benefits of creatine supplementation beyond those achieved by resistance training alone (59). Rapid weight loss, severe energy restriction, and the accompanying catabolic state may limit the capacity of any nutritional supplement to preserve or increase lean mass during this period. In contrast, interventions combining creatine with high-quality protein sources and other anabolic nutrients have reported improvements in appendicular lean mass, muscle strength, and functional outcomes (57, 60). These findings are consistent with the possibility that creatine and adequate amino acid availability address different components of the adaptive process: creatine supports cellular energy buffering and training capacity, whereas amino acids provide substrates and nutrient signals for tissue remodeling.

**Table 1:** Preclinical and clinical evidence on the enhancing effects of creatine combined with exercise in people with overweight or obesity.

Population	Intervention	Duration	Main outcomes	Results	Ref
Older men (n = 32) with overweight/obesity and MetS risk	Home-based resistance exercise + daily PA + multi-ingredient supplementation (collagen-based PLA vs. whey/casein + creatine, vitamin D, Ca, fish oil [M5])	3 months	Lean mass (TLM, ASM), LM/fat ratios, strength, performance, bone markers	Obesity and MetS strongly predicted blunted anabolic response. M5 group showed superior gains in lean mass, LM/fat ratios, strength, performance, and bone turnover vs. PLA	(57)
Women post-bariatric surgery (n = 15; BMI ≈ 39 kg/m²)	Resistance training + creatine (8 g/session) vs. placebo	8 weeks	Body weight, skeletal muscle mass, fat mass, strength	Both groups lost similar body weight and fat mass; muscle mass declined similarly; no between-group differences	(59)
Trained rats on standard or high-fat diet	Resistance training ± creatine monohydrate	8 weeks	Muscle performance, anabolic signaling	Creatine increased IGF-1 and p-AKT, but HF diet downregulated IGF-1/PI3K/Akt/mTOR proteins	(58)
Overweight older men (n = 15)	RET + HIIT ± whey protein, creatine, vitamin D, n-3 PUFA	21 weeks	MyoPS, lean mass	Supplementation did not significantly alter MyoPS post-training; unaccustomed MyoPS correlated with lean mass gains	(60)

Nevertheless, creatine should not be considered a continuously acting stimulator of MPS. Its better-established role is to increase phosphocreatine availability and support repeated high-intensity exercise, with downstream effects on training quality and adaptation (60). Thus, current evidence does not support a universal creatine strategy for obesity. Potential benefits are more plausibly determined by baseline muscle creatine availability, exercise stimulus, dietary adequacy, metabolic phenotype, and the clinical context of weight loss. These factors are particularly relevant when considering whether creatine should be combined with EAAs, because the two interventions may support different components of exercise-induced muscle remodeling.

### 6.2 Evidence and limitations of creatine–exercise interactions

Evidence for an interaction between creatine and exercise in obesity is encouraging but does not establish a universal synergistic effect. The majority of clinical investigations incorporated relatively modest sample sizes, often ranging from 15 to 32 participants, thereby limiting statistical power and increasing the likelihood that clinically meaningful between-group differences may remain undetected. Intervention durations of approximately 8–21 weeks may also be insufficient to characterize sustained changes in muscle mass, adipose remodeling, or long-term functional outcomes. Interpretation is further complicated by differences in study design, including retrospective analyses, heterogeneous exercise protocols, and the use of multi-ingredient supplements that prevent attribution of observed effects specifically to creatine. Studies conducted after bariatric surgery have often involved highly selected populations and relatively short follow-up, limiting generalizability. Preclinical studies provide useful mechanistic information but cannot by themselves establish clinical efficacy because of differences in species, creatine metabolism, dietary exposure, and exercise paradigms. A further limitation is that many studies compare creatine plus exercise with exercise alone but do not simultaneously characterize muscle protein turnover, phosphocreatine kinetics, metabolic phenotype, or inflammatory status. Consequently, it remains difficult to determine whether greater functional adaptation reflects improved training capacity, altered muscle bioenergetics, changes in protein turnover, or combinations of these processes. Future trials should therefore use factorial designs that directly compare exercise, creatine, EAA, and their combinations.

### 6.3 EAA supplementation with exercise: effects on muscle and adiposity

As discussed in Section 5, the principal rationale for combining EAAs with exercise is to provide essential amino acid substrates and nutrient signals during a period in which mechanical loading stimulates muscle remodeling. Leucine-sensitive mTORC1 signaling and increased amino acid availability can support MPS, but available clinical evidence does not consistently demonstrate large additional increases in absolute lean mass when adequate dietary protein is already provided (61, 62). Evidence for direct effects of EAA supplementation on adipose tissue is less established. Experimental studies suggest that amino acid availability can influence lipid metabolism and thermogenic pathways, but these observations should not be equated with demonstrated adipose remodeling in humans with obesity. The response to EAA supplementation is likely to depend on age, sex, obesity severity, sarcopenia status, baseline protein intake, energy balance, and exercise modality (63, 64). Older adults with sarcopenic or dynapenic obesity may exhibit greater anabolic resistance, whereas individuals with inadequate dietary protein may have more scope for benefit from targeted EAA provision. Conversely, when total protein intake is already sufficient, additional EAAs may provide relatively limited benefit beyond a well-designed exercise program.

Accordingly, functional outcomes may be more responsive than whole-body lean mass in some settings. Improvements in strength, walking performance, exercise tolerance, or other measures of physical function without substantial changes in lean mass suggest that adaptations may occur through neuromuscular and qualitative changes in muscle as well as through changes in tissue quantity (61, 63, 65, 66). Nutritional context is particularly important during weight loss. In hypocaloric interventions, EAA-containing formulations may help preserve muscle when endogenous protein availability is insufficient or when protein turnover is challenged by negative energy balance (67–69). However, the magnitude of benefit depends on the formulation, dose, timing, total dietary protein intake, and concomitant exercise stimulus. Overall, EAA supplementation appears most biologically relevant when amino acid availability is a limiting factor, particularly during energy restriction or when dietary protein intake is inadequate. Its combination with exercise is therefore best interpreted as potentially complementary rather than intrinsically synergistic (**Table 2**).

**Table 2:** Preclinical and clinical evidence on the enhancing effects of EAA combined with exercise in people with overweight or obesity.

Population	Intervention	Duration	Main outcomes	Results	Ref
People with overweight or obesity (n = 66; 50% female; age ≈ 37 yrs)	HIIT (2 d/wk) ± EAA (3.6 g twice daily)	8 weeks	Thigh lean mass, muscle CSA, muscle quality, whole-body protein turnover	HIIT and HIIT + EAA increased thigh LM, muscle size, and quality; EAA (with or without HIIT) increased protein synthesis, breakdown, and flux; women benefited more from HIIT + EAA	(62)
Elderly Japanese women with sarcopenic obesity (n = 139; >70 yrs)	Exercise ± EAA + catechin-fortified tea	3 months	Body composition, physical function, metabolic markers	Ex + N reduced total and trunk fat mass and improved walking parameters, vitamin D, and leptin; no significant gains in muscle mass or combined body composition indices	(63)
Older adults with dynapenic obesity (n = 48; BMI ≥ 30 kg/m² or high WC + low HGS)	Hypocaloric diet + RT ± EAA supplementation	5 months	Body composition, strength (1-RM, torque), physical performance	Both groups reduced body weight and fat mass and improved muscle strength and function; no between-group differences	(64)
People with overweight or obesity (n = 37; 51% female)	HIIT ± EAA (3.6 g twice daily)	8 weeks	Time to exhaustion, workload, HR, RPE	EAAs did not improve performance, fatigue resistance, or workload progression; reduced perceived exertion at 4 weeks in women only	(66)
Healthy overweight adults (n = 41; BMI 23–30 kg/m²)	Daily exercise + amino acid mixture (0.76 or 1.52 g/day) ± CLA (1.6 g/day), taken pre- and post-exercise	12 weeks	Waist and hip circumference, anthropometric indices	Amino acid–supplemented groups showed significant reductions in waist and hip circumferences vs. placebo	(69)
Untrained women (n = 21)	Aerobic + resistance training ± EAA (~18.3 g/day)	6 weeks	Body composition, strength, endurance	No changes in body composition or strength; EAA group improved treadmill time to exhaustion	(65)
Middle-aged men with severe obesity (BMI > 35 kg/m²; n = 40)	Low-calorie diet + structured physical activity ± protein, BCAA, or EAA mixture (PD-E07)	4 weeks	Muscle mass, body weight	PD-E07 group showed significant gains in muscle mass vs. control; other supplement groups showed no difference	(68)
Overweight adults (n = 200; 20–64 yrs)	A-mix 1,500 mg/day + promoted physical activity vs. placebo	12 weeks	Abdominal total, subcutaneous, and visceral fat	Significant reductions in abdominal total and subcutaneous fat in A-mix group vs. placebo	(67)
Healthy, independent older adults (n = 50; age 72 ± 1 yrs)	EAA 15 g/day ± supervised aerobic exercise 3x/week vs. placebo	24 weeks	Muscle protein synthesis, strength, body composition, VO_2_peak, walking speed	AE improved VO_2_peak and walking speed; EAA + AE increased muscle strength and EAA-stimulated protein synthesis; lean body mass unchanged	(61)

### 6.4 Methodological limitations of EAA–exercise studies

Evidence for EAA–exercise interactions remains difficult to compare across studies because of substantial methodological heterogeneity. Clinical investigations have frequently included small samples (approximately n = 21–66), although some larger cohorts have been reported. Differences in participant age, sex, obesity phenotype, sarcopenia status, baseline protein intake, and metabolic health complicate direct comparisons. Intervention duration has ranged from approximately 4 to 24 weeks, while EAA formulations, doses, timing, and accompanying dietary interventions have varied considerably. Such heterogeneity makes it difficult to determine whether differences in outcomes arise from amino acid composition, total protein intake, exercise exposure, energy restriction, or other concurrent interventions. The frequent use of mixed amino acid formulations also limits the ability to isolate the contribution of individual EAAs or to determine whether a specific formulation is superior to adequate dietary protein. These limitations are particularly important for interpreting claims of synergy. A true interaction requires direct comparison of the individual interventions with their combination under otherwise comparable conditions. Many existing studies do not provide this experimental structure. Thus, the available evidence supports biological complementarity but is insufficient to establish a reproducible EAA–exercise synergy in obesity.

### 6.5 Integrated effects of exercise, creatine, and EAAs on the muscle–adipose–brain axis

The preceding sections establish exercise as the principal physiological stimulus for remodeling the muscle–adipose–brain network, while creatine and EAAs may provide complementary nutritional support for different components of this adaptation. The key question is therefore not whether either supplement independently regulates the entire axis, but whether their distinct effects on muscle bioenergetics and protein turnover could indirectly strengthen exercise-responsive communication with adipose tissue and the brain. Exercise-induced signaling provides the most established entry point into this network. Contracting skeletal muscle releases myokines and metabolites, including irisin-related signals, meteorin-like (Metrnl), lactate, and cathepsin B, that can influence adipose metabolism, immune activity, and brain function (70–74). For example, experimental evidence links the PGC-1α/FNDC5/irisin pathway to adipose thermogenic programs and hippocampal BDNF signaling, whereas Metrnl has been implicated in eosinophil/IL-4-dependent immune remodeling and beige adipocyte formation (72–74). Lactate and cathepsin B have likewise been associated with hippocampal BDNF-related responses and neuroplasticity in experimental models (70, 71). These pathways provide plausible mechanisms through which the exercise stimulus can extend beyond skeletal muscle, but their quantitative contribution in humans with obesity remains incompletely defined.

Creatine could potentially influence this network upstream by improving the energetic capacity of skeletal muscle during repeated exercise. Increased phosphocreatine availability may support force production and training volume, thereby indirectly increasing the magnitude or consistency of exercise-induced adaptations. EAAs could act at a complementary point by providing substrates and nutrient signals required for exercise-induced protein remodeling. In this framework, creatine and EAAs are not assumed to activate the same pathway or to produce a molecularly synergistic response. Rather, creatine may primarily support the energetic component of training, whereas EAAs support the substrate and nutrient-sensing component of muscle remodeling. The potential downstream consequences for adipose tissue are more uncertain. Exercise can influence adipose tissue through sympathetic signaling, myokines, metabolites, and immune remodeling. Experimental studies involving Metrnl, beige adipocytes, leptin–BDNF signaling, and neuro-adipose communication indicate that adipose tissue is responsive to both endocrine and neural signals (72, 75, 76). However, there is currently insufficient evidence to conclude that creatine or isolated EAA supplementation directly enhances these exercise-induced adipose pathways in humans with obesity. Preclinical observations suggesting effects on thermogenesis, lipid metabolism, or adipose inflammation should therefore be regarded as mechanistic hypotheses rather than established clinical outcomes.

A similar evidence gradient applies to the brain. Exercise-responsive metabolites and myokines can influence neurotrophic and metabolic pathways, while creatine has a more direct mechanistic rationale for supporting cellular and cerebral energy buffering. EAAs may additionally influence neurotransmitter precursor availability and nutrient-sensitive signaling, particularly through tryptophan and large neutral amino acid transport. Nevertheless, direct studies simultaneously assessing exercise, creatine, EAA availability, adipose signaling, neuroinflammation, and neurobehavioral outcomes in obesity are scarce. Therefore, a causal chain from supplementation to improved adipose–brain communication or reduced neuroinflammation cannot currently be inferred. The integrated model should consequently be considered a graded evidence framework rather than an established synergistic pathway. Human evidence is strongest for exercise-induced muscle adaptation and for creatine- or EAA-associated effects on selected muscle and functional outcomes. Evidence linking exercise to circulating myokines, metabolites, adipose remodeling, and brain signaling is supported by a combination of human and preclinical studies, whereas direct evidence that creatine plus EAAs amplify these inter-organ effects in obesity remains limited. This distinction is essential for avoiding overinterpretation of mechanistic findings. Accordingly, future trials should directly test whether adding creatine and EAAs to standardized exercise produces effects beyond exercise alone and whether these effects are mediated by measurable changes in muscle bioenergetics, protein turnover, circulating metabolites, adipokines/myokines, immune-cell phenotypes, neuroinflammatory markers, or brain function. A factorial design incorporating exercise, creatine, EAA, and combined supplementation would be particularly informative. Such studies could determine whether the proposed interaction is genuinely synergistic, merely additive, or primarily attributable to improved exercise adherence and training capacity (**Table 3**).

**Table 3:** Exercise-induced mediators linking skeletal muscle, adipose tissue and brain.

Exercise-induced mediator	Primary source	Primary target(s)	Major biological actions	Role in muscle–adipose–brain crosstalk	Ref
Irisin (FNDC5)	Contracting skeletal muscle	White adipose tissue	Induces browning of white adipose tissue by increasing PPAR-α and UCP1 expression, mitochondrial biogenesis, oxygen consumption, and energy expenditure	Serves as a key endocrine myokine linking skeletal muscle activity to adipose tissue remodeling and metabolic homeostasis	(74)
PGC-1α/FNDC5–BDNF axis	Skeletal muscle and hippocampal neurons	Brain (hippocampus)	Increases BDNF expression, promotes synaptic plasticity, neuronal survival, and neuroprotection	Establishes a direct molecular connection between exercise-induced muscle signaling and brain plasticity	(73)
Meteorin-like (Metrnl)	Exercised skeletal muscle	Adipose tissue immune cells	Stimulates eosinophil-derived IL-4 production, alternative (M2) macrophage activation, beige adipocyte formation, thermogenesis, and glucose homeostasis	Couples skeletal muscle with adipose immunometabolism through immune-mediated endocrine signaling	(72)
Sympathetic neuro-adipose signaling (Leptin pathway)	Hypothalamus via sympathetic nerves	White adipose tissue	Activates hormone-sensitive lipase, stimulates lipolysis, and regulates adipocyte metabolism through neuro-adipose junctions	Demonstrates top-down neural regulation of adipose tissue by the brain during energy homeostasis	(76)
Cathepsin B (CTSB)	Exercised skeletal muscle	Brain (hippocampus)	Increases BDNF and doublecortin (DCX) expression, enhances neurogenesis, learning, and memory	Identifies a circulating exercise-induced myokine that links skeletal muscle activity with cognitive function	(70)
Lactate	Contracting skeletal muscle	Brain (hippocampus)	Crosses the blood–brain barrier, activates the SIRT1/PGC-1α/FNDC5 pathway, induces BDNF expression, and enhances learning and memory	Functions as a metabolic messenger transmitting exercise signals from muscle to the brain	(71)
Adipose-derived macrophage trafficking	Visceral adipose tissue	Hypothalamus	Promotes infiltration of metabolically activated macrophages into the hypothalamus, leading to neuroinflammation and neuronal dysfunction	Demonstrates pathological adipose-to-brain immune communication during obesity	(79)
Leptin–BDNF neural pathway	Hypothalamus	White and brown adipose tissue	Regulates sympathetic innervation, thermogenesis, lipolysis, and adipose tissue plasticity	Defines a central neuroendocrine circuit controlling adipose tissue remodeling and whole-body energy balance	(75)
Neuronal Growth Regulator-1 (NEGR1)	Adipose tissue	Sympathetic neurons	Maintains sympathetic neurite integrity, preserves adipose innervation, and supports neuronal survival	Reveals a novel adipose-to-neuronal signaling mechanism disrupted in obesity	(80)
Beige adipocyte-derived IL-4 signaling	Beige adipose tissue	Brain (microglia and hippocampus)	Suppresses neuroinflammation, restores hippocampal synaptic plasticity, and improves cognitive performance through IL-4-dependent mechanisms	Demonstrates that metabolically healthy adipose tissue exerts neuroprotective effects via immune–brain communication	(9)

## 7 Muscle–adipose–brain crosstalk, immunometabolism, and neurobehavioral outcomes

### 7.1 Immunometabolic communication and circulating metabolites in obesity

Immunometabolism has emerged as a central mechanism linking obesity-associated metabolic dysfunction with altered immune and neural function. Rather than representing a simple increase in inflammatory signaling, obesity induces metabolic remodeling of peripheral and central immune cells, including changes in substrate utilization, mitochondrial function, redox balance, and inflammatory signaling. These changes can create reciprocal interactions between metabolic tissues and immune cells, whereby nutrient excess and metabolic stress alter immune-cell function, while immune mediators further impair insulin sensitivity, tissue metabolism, and neuronal function. The hypothalamus is particularly sensitive to these alterations. Hypothalamic microglia respond early to nutritional excess and undergo dynamic changes in metabolic state, mitochondrial function, environmental sensing, and circadian regulation (77, 78). Importantly, these responses are not necessarily equivalent to a uniform pro-inflammatory phenotype; rather, obesity may induce metabolically adapted immune states whose functions vary with disease duration and tissue context. Visceral adipose tissue can contribute to this process by releasing inflammatory mediators and by providing metabolically activated macrophages that accumulate within hypothalamic regions during obesity (79). Thus, central immunometabolic dysfunction can be viewed as partly embedded within broader peripheral tissue–immune communication. Adipose tissue also participates in bidirectional neuroimmune regulation. Hypothalamic leptin signaling contributes to the maintenance of sympathetic innervation of adipose tissue through BDNF-dependent pathways, thereby influencing thermogenesis, lipolysis, and metabolic flexibility (75). Obesity can disrupt this regulatory circuit, while altered adipose-derived signals such as NEGR1 may further compromise sympathetic neurite integrity and adipose–neural communication (80). Conversely, metabolically competent beige adipose tissue may exert anti-inflammatory effects through IL-4-dependent pathways that influence microglial activity and hippocampal function (9). These findings suggest that adipose tissue quality, rather than adipose mass alone, may influence neuroimmune homeostasis.

Circulating metabolites provide an additional mechanism through which muscle and adipose tissue may communicate with immune cells. A recent metabolomic and lipidomic study involving two human cohorts identified reduced circulating sarcosine concentrations in individuals with sarcopenia. In complementary mouse experiments, sarcosine administration enhanced muscle regeneration and adipose thermogenesis and promoted anti-inflammatory macrophage polarization through activation of the GCN2 pathway (81). This study is particularly relevant because it links a circulating metabolite to coordinated changes in muscle, adipose tissue, and immune-cell phenotype. However, the interventional findings were obtained primarily in mice, and the study addressed sarcopenia rather than obesity; therefore, it should be regarded as an emerging mechanistic example rather than evidence that sarcosine or related metabolic pathways mediate the effects of creatine, EAAs, or exercise in obesity. This metabolite-centered perspective broadens the muscle–adipose–brain framework beyond classical cytokines and myokines. Exercise can alter circulating metabolites and myokines, whereas changes in muscle bioenergetics, adipose metabolism, and nutritional substrate availability may modify the biochemical environment encountered by immune cells. Whether creatine or EAA supplementation changes such circulating immunometabolic mediators in obesity remains largely unexplored. This represents an important mechanistic gap because metabolite profiling combined with immune-cell phenotyping could help determine whether nutritional supplementation modifies inter-organ communication indirectly through immune-cell reprogramming.

### 7.2 Neuroinflammation and mitochondrial dysfunction

Neuroinflammation in obesity is increasingly understood as a metabolically regulated process rather than an isolated inflammatory response. Chronic nutrient excess, insulin resistance, altered lipid metabolism, oxidative stress, and mitochondrial dysfunction can modify the interactions among microglia, astrocytes, neurons, and peripheral immune cells. High-fat feeding, for example, can alter microglial substrate utilization, mitochondrial bioenergetics, and circadian regulation (77, 78). These observations suggest that the functional state of neural immune cells depends not only on inflammatory signals but also on the metabolic environment in which they operate. Peripheral metabolic tissues can reinforce this process. Visceral adipose tissue contributes inflammatory mediators and immune cells to the systemic environment and has been linked to hypothalamic immune activation (79). At the same time, disruption of leptin–BDNF signaling and adipose-derived neurotrophic pathways may impair sympathetic regulation of adipose tissue (75, 80). The resulting feedback between adipose dysfunction, altered autonomic signaling, and central immune activation provides a plausible mechanism through which peripheral metabolic dysfunction can influence brain physiology. Exercise provides a physiological counter-regulatory stimulus. Exercise-responsive pathways involving PGC-1α/FNDC5/irisin, lactate, and cathepsin B have been associated with hippocampal BDNF expression, neurogenesis, and synaptic plasticity (70, 71, 73). However, the extent to which these pathways account for clinically meaningful improvements in neuroinflammation in humans with obesity remains uncertain. Similarly, creatine has a mechanistic rationale for supporting cerebral phosphocreatine and ATP buffering (40–43, 82), whereas EAAs can influence amino acid availability and neurotransmitter-related metabolism (53, 55). Direct evidence that either supplement reduces obesity-associated neuroinflammation, however, remains limited. Accordingly, exercise, creatine, and EAAs should currently be viewed as potentially complementary interventions whose effects on neuroimmune function require direct experimental confirmation. Future studies should simultaneously measure circulating metabolites, immune-cell phenotypes, inflammatory mediators, mitochondrial function, and neurobehavioral outcomes rather than inferring changes in neuroinflammation from peripheral metabolic improvements alone.

### 7.3 Depression and mood-related outcomes

Depressive symptoms are more prevalent among individuals with obesity, and their association may involve overlapping metabolic, inflammatory, and psychosocial mechanisms. From a biological perspective, obesity-associated inflammation, insulin resistance, mitochondrial dysfunction, and altered cerebral energy metabolism may contribute to vulnerability to depressive symptoms. However, these mechanisms are multifactorial, and obesity should not be considered to cause depression through a single muscle–adipose–brain pathway. Cerebral bioenergetics provides one potentially relevant mechanism. Studies in major depressive disorder have reported alterations in phosphocreatine and ATP-related metabolism, providing a rationale for investigating creatine as an adjunctive intervention. Clinical trials have reported beneficial effects of adjunctive creatine in some depressive disorders, including when combined with selective serotonin reuptake inhibitors, while changes in cerebral phosphocreatine have been associated with treatment response (40, 41). Evidence also exists for adjunctive creatine in bipolar depression, although results remain heterogeneous and these populations differ substantially from individuals with obesity (82, 83). Amino acid availability may influence mood-related neurobiology through precursor supply for neurotransmitter synthesis. Tryptophan competes with other large neutral amino acids for transport across the blood–brain barrier, and dietary manipulation can modify the plasma tryptophan-to-LNAA ratio (53). Studies using tryptophan-rich proteins such as α-lactalbumin have demonstrated changes in this ratio and selected cognitive or stress-related outcomes (55). These findings provide biological plausibility for amino acid–dependent modulation of brain function, but they do not establish that EAA supplementation prevents or treats depression in obesity. Exercise may provide an additional pathway through which peripheral and central adaptations interact. Exercise-induced signaling involving irisin, lactate, cathepsin B, and BDNF has been associated with neuroplasticity and mood-related adaptations (71, 73). Nevertheless, the direct contribution of creatine or EAA supplementation to these exercise-induced neurobehavioral responses in obesity remains largely untested. Overall, current evidence supports a plausible relationship among metabolic dysfunction, cerebral bioenergetics, inflammation, and mood, but direct evidence for a creatine–EAA–exercise intervention in obesity-associated depression is insufficient. Depression should therefore be considered an important clinical endpoint for future trials rather than an established therapeutic outcome of the combined intervention.

### 7.4 Cognitive function

Cognitive impairment in obesity may involve changes in memory, executive function, attention, processing speed, and cognitive flexibility. Potential contributors include insulin resistance, vascular dysfunction, chronic inflammation, altered cerebral energy metabolism, and impaired neuroplasticity. These mechanisms overlap with the metabolic and neuroimmune disturbances discussed above but should be distinguished from evidence directly demonstrating cognitive effects of creatine or EAA supplementation in obesity. Brain energy metabolism is one potential target of creatine supplementation. Creatine increases cerebral phosphocreatine availability and has been associated with improvements in selected cognitive domains, particularly under conditions of increased energetic demand such as fatigue or hypoxia (42, 43). Cognitive effects have also been reported in some psychiatric populations receiving adjunctive creatine (83). However, these findings cannot be directly extrapolated to obesity, in which the clinical evidence remains limited. Amino acid availability may influence cognition through neurotransmitter precursor pathways. Changes in the plasma tryptophan-to-LNAA ratio can affect cerebral tryptophan availability and serotonergic signaling, while tryptophan-rich protein interventions have been associated with selected cognitive responses under stress (53, 55). Nevertheless, there is currently insufficient evidence to conclude that isolated EAA supplementation improves cognitive function in obesity. Exercise provides a more established behavioral stimulus for neuroplastic adaptation. Exercise-responsive myokines and metabolites, including irisin-related signals, lactate, and cathepsin B, have been linked to BDNF signaling, hippocampal plasticity, and neurogenesis (70, 71, 73). Whether creatine or EAAs amplify these responses remains an open question. Future studies should therefore incorporate validated cognitive assessments alongside metabolic, inflammatory, and neuroimaging measures rather than relying solely on changes in body composition as indicators of brain benefit.

### 7.5 Integrated neurobehavioral and brain-health implications

Brain health represents an important downstream outcome of the metabolic and inter-organ processes discussed throughout this review. In obesity, alterations in systemic metabolism, adipose tissue biology, immune signaling, and cerebral bioenergetics may converge to influence neuroinflammation, cognition, and mood. The proposed role of exercise, creatine, and EAAs should nevertheless be interpreted according to the strength of available evidence. Exercise has the strongest rationale as the primary physiological stimulus and can influence muscle-derived signaling, systemic metabolism, and neuroplasticity. Creatine may provide additional support for muscle and cerebral energy buffering, whereas EAAs primarily support amino acid availability, protein turnover, and nutrient-sensitive signaling. These actions are biologically complementary, but their ability to jointly improve brain outcomes in obesity has not been established in adequately powered clinical trials. A major knowledge gap is therefore the absence of integrated intervention studies linking changes in muscle and adipose tissue to immune-cell phenotypes and clinically meaningful brain outcomes. Future trials should combine body-composition and functional measurements with circulating metabolomics, myokine/adipokine profiling, immune-cell phenotyping, inflammatory and mitochondrial biomarkers, and validated assessments of cognition and depressive symptoms. Where feasible, neuroimaging could provide an additional bridge between peripheral metabolic adaptation and central nervous system function. Importantly, improvements in brain-related outcomes should not be inferred solely from weight loss or changes in circulating inflammatory markers. Demonstrating a muscle–adipose–brain effect will require longitudinal studies showing that an intervention changes peripheral metabolic or immune signals and that these changes are associated with corresponding alterations in brain function or neurobehavioral outcomes. Taken together, current evidence supports a conceptual model in which exercise is the principal driver of muscle–adipose–brain remodeling, with creatine and EAAs representing potentially complementary nutritional strategies (**Table 4**). The strongest evidence concerns muscle bioenergetics, protein metabolism, and physical function; evidence for adipose–immune and brain effects is more heterogeneous, and direct evidence for improvements in obesity-associated neuroinflammation, depression, or cognition remains limited. This evidence hierarchy should guide the design of future precision-oriented trials rather than serve as a basis for claiming established neuroprotective or antidepressant effects of combined supplementation.

**Table 4:** Potential mechanisms through which creatine, essential amino acids, and exercise influence depression, cognitive function, and brain health in obesity.

Mechanistic pathway	Obesity-associated alteration	Potential role of creatine	Potential role of essential amino acids	Contribution of exercise	Ref
Brain bioenergetics	Impaired mitochondrial function, reduced phosphocreatine availability, decreased ATP production	Increases brain phosphocreatine stores, enhances ATP buffering, improves mitochondrial bioenergetics	Supports mitochondrial protein turnover and cellular energy metabolism	Promotes mitochondrial biogenesis and oxidative metabolism	(40–43, 82, 83)
Neurotransmitter synthesis	Impaired serotonin synthesis and neurotransmitter homeostasis	Indirectly supports neurotransmission by improving neuronal energy availability	Provides tryptophan and other EAAs required for serotonin and catecholamine synthesis	Enhances neurotransmitter turnover and synaptic function	(53, 55)
Neuroplasticity (BDNF signaling)	Reduced BDNF expression, impaired synaptic plasticity, and hippocampal dysfunction	May indirectly enhance neuroplasticity by improving neuronal bioenergetics	Supports synthesis of proteins required for synaptic remodeling	Activates the PGC-1α/FNDC5/irisin pathway, increasing hippocampal BDNF expression	(71, 73)
Muscle–brain communication	Reduced secretion of exercise-responsive myokines in obesity	Enhances exercise capacity, potentially augmenting myokine release	Preserves skeletal muscle mass and anabolic responsiveness	Stimulates release of irisin, lactate, and cathepsin B that promote neuroplasticity	(71, 73)
Adipose–brain communication	Increased adipose inflammation, macrophage infiltration, impaired sympathetic regulation	May indirectly attenuate systemic inflammation through improved metabolic function	May improve adipose tissue metabolism and metabolic flexibility	Promotes adipose tissue remodeling and anti-inflammatory signaling	(9, 75, 79, 80)
Microglial immunometabolism	Microglial metabolic reprogramming, circadian disruption, oxidative stress	Supports cellular energy homeostasis and may reduce oxidative stress	Supports immune cell metabolism through amino acid availability	Attenuates neuroinflammation and improves immune regulation	(77, 78, 96)
Oxidative stress	Increased reactive oxygen species and mitochondrial dysfunction	Improves mitochondrial energy buffering and reduces oxidative stress	Provides amino acid substrates required for antioxidant defense and glutathione synthesis	Upregulates endogenous antioxidant systems	(41, 96)
Depression	Neuroinflammation, impaired cerebral bioenergetics, reduced neuroplasticity	Improves depressive symptoms as adjunctive therapy and increases brain phosphocreatine	May support mood regulation through serotonin precursor availability (limited evidence)	Improves mood through neurotrophic, metabolic, and anti-inflammatory mechanisms	(40, 41, 53, 55, 82, 83)
Cognitive function	Impaired memory, executive function, attention, and processing speed	Improves working memory, reasoning ability, attention, and verbal fluency	Enhances cognition under stress by increasing plasma tryptophan availability	Improves learning, memory, and hippocampal function	(42, 43, 55, 70, 71, 73, 83)
Overall brain health	Progressive neuronal dysfunction, reduced resilience, and impaired brain–body communication	Supports long-term neuronal energy homeostasis and cellular resilience	Maintains protein turnover and provides precursors for neurotransmitter synthesis	Integrates neuroplastic, metabolic, anti-inflammatory, and endocrine adaptations	(9, 40–43, 53, 55, 70, 71, 73, 75, 77–80, 82, 83, 96)

## 8 Translational priorities and precision management

Obesity is biologically and clinically heterogeneous, and this heterogeneity may contribute to substantial variation in responses to exercise and nutritional interventions (84–86). In the context of the present review, a precision-oriented framework should therefore not imply that creatine or EAA supplementation is already an established individualized therapy. Rather, the immediate translational objective is to determine whether these nutritional strategies provide reproducible benefits when combined with exercise and to identify the clinical and biological characteristics that may modify treatment response.

### 8.1 Clinical efficacy and clinically meaningful outcomes

The first translational priority is to establish the clinical efficacy of exercise combined with creatine and/or EAA supplementation. Current evidence supports exercise as the principal intervention for improving physical function and body composition, whereas nutritional supplementation may provide additional support under selected conditions. However, direct evidence for a combined creatine–EAA–exercise intervention in obesity remains limited. Precision management should therefore begin with adequately powered randomized controlled trials that compare exercise alone with exercise plus creatine, exercise plus EAAs, and their combined use. Clinically meaningful outcomes should extend beyond body weight and include changes in fat mass, lean mass, muscle strength, physical function, and metabolic health. Neurobehavioral outcomes, including cognition and mood, may also be informative, but should initially be considered secondary or exploratory endpoints unless direct effects are demonstrated. The broader precision-nutrition literature supports the concept that individual metabolic characteristics can modify responses to nutritional interventions, but this principle should not be interpreted as evidence that creatine or EAA supplementation has already been validated as a personalized treatment for obesity (84–86).

### 8.2 Dose, timing, and exercise phenotype

A second priority is to determine how supplementation dose, timing, duration, and formulation interact with exercise phenotype. Creatine and EAAs have distinct biological roles, and their potential benefits are unlikely to be independent of the exercise stimulus. Resistance exercise may be particularly relevant when the primary objective is preservation or gain of skeletal muscle, whereas aerobic or combined exercise may be more important for whole-body metabolic adaptation. Future studies should therefore examine supplementation within clearly defined exercise protocols and account for baseline dietary protein intake, energy availability, physical activity, and metabolic phenotype. Factorial designs comparing exercise, creatine, EAA, and combined interventions would be particularly useful for determining whether the observed effects are additive, independent, or interactive. Importantly, such designs would also prevent the assumption of biological synergy when direct evidence for an interaction is unavailable.

### 8.3 Responders, non-responders, and obesity heterogeneity

A third priority is to characterize responders and non-responders using prospectively defined biological and clinical characteristics. Potential modifiers include baseline skeletal muscle mass and quality, visceral adiposity, metabolic health, habitual protein intake, training status, age, sex, comorbidities, medication use, and inflammatory phenotype (84–88). Baseline creatine availability and amino-acid status may also be relevant to the magnitude of adaptation, although their value as predictive biomarkers require prospective validation. Sex should also be considered when investigating heterogeneity in treatment response because males and females differ in body-fat distribution, skeletal muscle characteristics, endocrine regulation, and metabolic responses to exercise (89, 90). Nevertheless, available evidence does not justify universal sex-specific creatine or EAA protocols. Instead, future trials should be adequately powered to determine whether treatment effects differ by sex and across relevant physiological stages. Genetic and nutrigenetic characteristics may provide additional information about interindividual variability. Although variants related to nutrient metabolism, energy regulation, and exercise adaptation have been investigated in the context of personalized nutrition and sports performance (87, 88), their ability to predict responses specifically to creatine or EAA supplementation in obesity remains uncertain. Accordingly, genetic information should currently be regarded as a research tool for response stratification rather than a basis for routine supplementation decisions. The gut microbiome represents another potentially relevant source of interindividual variation. Microbial communities and their metabolites can influence host metabolism, immune signaling, and nutrient processing and may therefore contribute to differences in metabolic responses among individuals with obesity (91, 92). However, evidence directly linking baseline microbiome composition to the response to creatine or EAA supplementation in obesity remains insufficient. The microbiome should consequently be considered an investigational modifier rather than an established criterion for selecting supplementation strategies.

### 8.4 Biomarker-guided mechanistic stratification

A fourth priority is to identify biomarkers that connect clinical outcomes with the biological mechanisms proposed in this review. Circulating metabolites, amino-acid profiles, myokines, adipokines, inflammatory mediators, and markers of mitochondrial function could help determine whether exercise and nutritional supplementation modify immunometabolic communication among skeletal muscle, adipose tissue, and the brain. Multi-omics approaches integrating metabolomics, lipidomics, proteomics, and transcriptomics may facilitate the identification of biological signatures associated with anabolic resistance, altered substrate metabolism, inflammation, or mitochondrial dysfunction (93). Such approaches may also help distinguish baseline predictors of treatment response from biomarkers that emerge as a consequence of successful intervention. Nevertheless, these approaches should initially be used for mechanistic characterization and hypothesis generation; their predictive value must be established prospectively before they can be used to guide individualized supplementation.

### 8.5 Multi-omics, neuroimaging, and digital/AI-enabled approaches

At a later translational stage, integration of multi-omics data with body-composition measurements, neuroimaging, physical-activity data, and longitudinal clinical phenotyping may provide a more comprehensive characterization of treatment response. Multi-omics frameworks have already been proposed as a potential approach for improving biological stratification in precision obesity management, although important methodological and validation challenges remain (84, 93). Artificial intelligence and machine-learning approaches may subsequently help integrate high-dimensional clinical, behavioral, imaging, and molecular datasets to identify patterns associated with treatment response (94). However, such models require external validation, transparent reporting, and prospective clinical testing before they can be used to determine individualized creatine or EAA supplementation. Digital health tools and wearable technologies may also facilitate longitudinal monitoring of adherence, physical activity, and physiological adaptation, but their role in optimizing supplementation remains investigational.

Neuroimaging may be particularly informative for testing whether peripheral metabolic and inflammatory adaptations are accompanied by measurable changes in brain structure or function. When combined with circulating biomarkers, metabolomics, body-composition assessment, and standardized neuropsychological outcomes, these approaches could help determine whether changes in the muscle–adipose–brain axis translate into clinically meaningful brain-related outcomes. Overall, precision management should be considered a stepwise translational process rather than an established clinical application of creatine and EAA supplementation. The immediate priority is to determine whether these interventions provide meaningful benefits beyond exercise alone. Subsequent research should identify how dose, exercise phenotype, baseline biological characteristics, and biomarkers modify responsiveness. Only after these relationships have been prospectively validated should multi-omics and AI-based approaches be considered for individualized intervention algorithms. This framework maintains exercise as the central physiological stimulus while positioning creatine and EAAs as potentially complementary nutritional strategies whose benefits require further clinical validation.

## 9 Metabolic and clinical implications of exercise with creatine and EAA supplementation

### 9.1 Fat mass reduction and adipose tissue remodeling

Exercise remains the primary intervention for improving body composition in individuals with overweight and obesity, with resistance, aerobic, and high-intensity interval training producing distinct but partially overlapping effects on energy expenditure, adiposity, and metabolic function (67, 69) (**Figure 2**). Nutritional supplementation may complement these adaptations by supporting the preservation of metabolically active lean tissue during energy restriction or weight-loss interventions. EAA supplementation can provide essential substrates and anabolic signals for muscle protein synthesis, whereas creatine primarily supports phosphocreatine-dependent energy buffering and exercise capacity. Accordingly, their potential clinical value may lie less in directly increasing fat loss and more in facilitating the preservation of lean mass while exercise reduces adiposity. Evidence that specific amino-acid combinations directly augment exercise-induced fat loss in humans remains limited and heterogeneous. Although amino acids such as lysine, arginine, and phenylalanine have been investigated in relation to lipid metabolism and body composition (69), these findings should not be interpreted as evidence that EAA supplementation consistently produces additional fat loss when combined with exercise. Similarly, creatine supplementation does not appear to promote fat accumulation and, when combined with resistance exercise, may contribute to favorable changes in lean mass and body composition (6). Mechanistic and preclinical studies also suggest that creatine metabolism may influence adipose tissue energy expenditure and thermogenic pathways, but the extent to which these mechanisms translate into clinically meaningful reductions in adiposity in humans remains uncertain (31, 95). From a clinical perspective, therefore, the principal objective should be improvement in the ratio of functional lean tissue to adipose tissue rather than weight loss alone. This distinction is particularly relevant in obesity, where preservation of skeletal muscle during caloric restriction may help maintain physical function and metabolic capacity. Exercise combined with appropriate nutritional support may consequently represent a more clinically meaningful strategy than interventions focused exclusively on reducing body weight.

**Figure 2:**
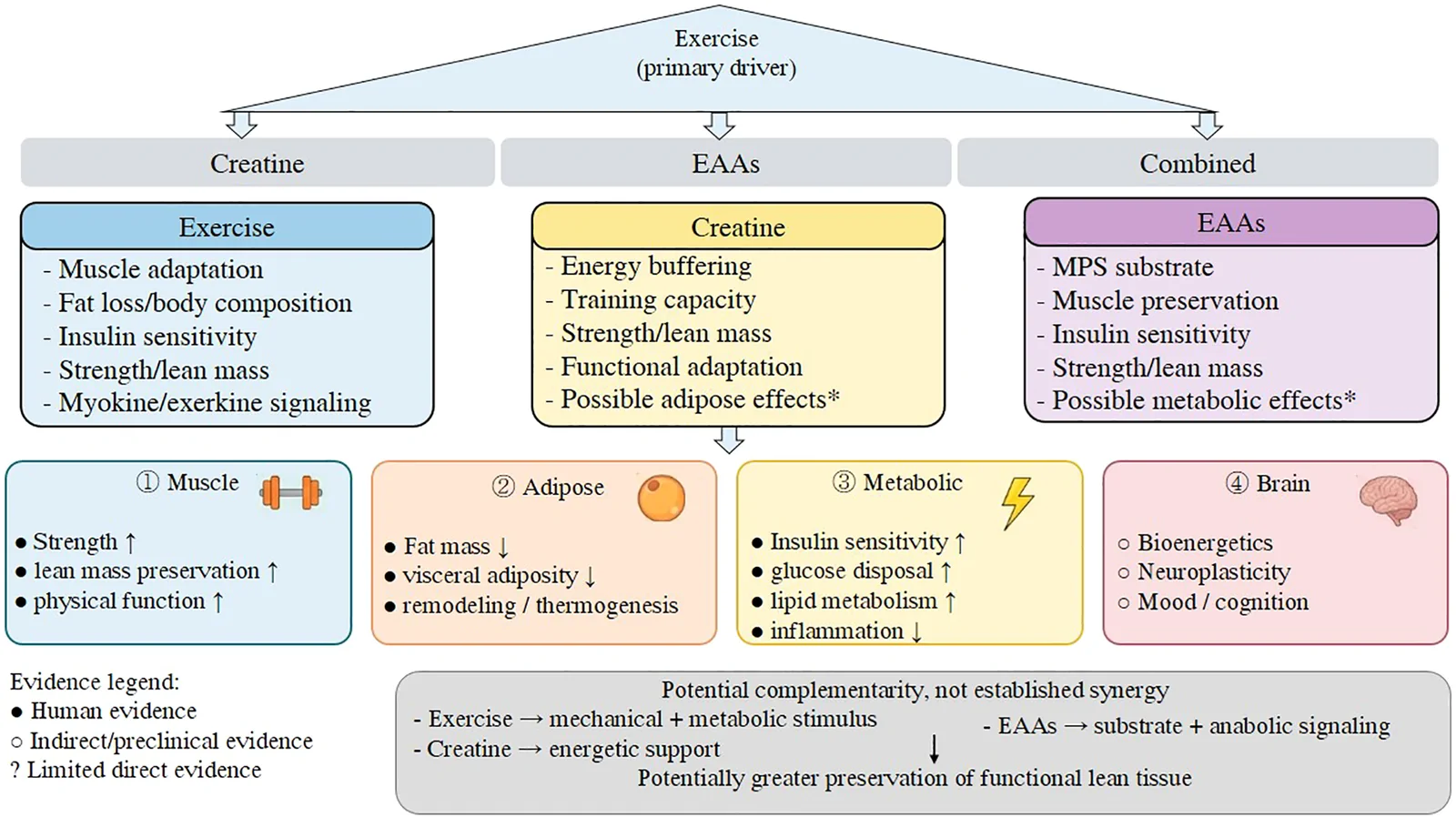
Distinct and potentially complementary effects of exercise, creatine, and essential amino acids on metabolic and clinical outcomes in obesity. Exercise, creatine, and EAAs contribute through partially distinct mechanisms. Exercise provides the principal mechanical and metabolic stimulus for skeletal muscle adaptation, energy expenditure, adipose remodeling, and improved glucose and lipid metabolism. Creatine primarily supports phosphocreatine-dependent energy buffering, exercise capacity, strength, and lean-mass adaptation, particularly when combined with resistance exercise. EAAs provide anabolic substrates and nutrient signals that support muscle protein synthesis and preservation of lean tissue, especially when protein or energy availability is limited. These interventions may therefore converge on outcomes such as muscle strength, physical function, body composition, and metabolic health. Effects on adipose tissue inflammation, thermogenesis, and brain-related outcomes remain less firmly established and are represented as emerging or limited evidence. The overall model distinguishes established human-supported outcomes from proposed or indirect mechanisms and indicates that the combination of creatine, EAAs, and exercise should currently be viewed as potentially complementary rather than definitively synergistic.

### 9.2 Improvements in insulin sensitivity, inflammation, and metabolic health

Exercise can improve insulin sensitivity, glucose disposal, mitochondrial function, and lipid metabolism, while preservation or restoration of skeletal muscle mass may further support whole-body glucose handling (60, 62, 66). EAA supplementation may complement these effects by stimulating muscle protein synthesis and providing amino-acid substrates required for tissue remodeling, although direct evidence for additional improvements in insulin sensitivity specifically attributable to EAA supplementation in obesity remains limited. In this context, the metabolic effects of EAAs should be distinguished from those of exercise, which represents the principal stimulus for improving metabolic flexibility. Creatine may provide an additional, mechanistically distinct contribution through its effects on cellular energy buffering and exercise performance. By increasing intramuscular phosphocreatine availability, creatine can support repeated high-intensity contractions and potentially facilitate greater training volume or quality (6, 31). Any subsequent improvements in glucose regulation or metabolic health are therefore more plausibly interpreted as partly mediated by enhanced training adaptation rather than as a direct pharmacological effect of creatine. Combined exercise and nutritional interventions may also influence systemic inflammatory status, but the magnitude and consistency of changes in circulating inflammatory biomarkers such as IL-6 and CRP vary across populations, exercise modalities, intervention durations, and nutritional formulations (60, 66). Accordingly, reductions in inflammation should be regarded as a potential downstream consequence of improved metabolic health, adiposity, and physical activity rather than an established direct effect of combined creatine–EAA supplementation. This distinction is important because obesity-related inflammation is heterogeneous and involves interactions among adipose tissue, skeletal muscle, immune cells, and the brain. Overall, the strongest clinical rationale for integrating exercise with creatine and/or EAA supplementation is the simultaneous targeting of muscle function, body composition, and metabolic health. Whether combining creatine and EAAs produces effects that exceed those achieved by either intervention with exercise alone remains an important unanswered question and requires appropriately powered factorial randomized trials.

### 9.3 Potential applications in sarcopenic obesity and weight management

Sarcopenic obesity presents a particularly relevant clinical context because excess adiposity coexists with reduced muscle mass, muscle quality, or physical function, thereby increasing both metabolic and functional risk (57, 68). In this population, weight-loss strategies that reduce fat mass without adequately preserving skeletal muscle may be clinically suboptimal. Resistance exercise is therefore particularly important for maintaining or increasing muscle strength and lean tissue, whereas aerobic exercise can contribute to improvements in cardiorespiratory fitness and reductions in adiposity (57, 67). Protein or EAA supplementation may further support muscle protein synthesis and the preservation of lean tissue, particularly when energy intake is restricted or anabolic responsiveness is impaired (57, 68). However, the evidence base for nutritional supplementation specifically in sarcopenic obesity remains relatively limited and heterogeneous. Recent evidence syntheses indicate that resistance training can improve body fat, muscle mass, strength, and physical function in people with sarcopenic obesity, while combining exercise with increased protein intake may provide additional benefits for body fat reduction; nevertheless, the available studies are too heterogeneous to establish a specific supplementation protocol for clinical practice (57, 68). Creatine may be particularly relevant when preservation of strength and functional capacity is a primary therapeutic objective, because its principal established role is to support muscle energy availability and training adaptation rather than directly induce weight loss (6). Nevertheless, evidence specifically testing creatine plus EAA supplementation together with exercise in well-defined sarcopenic-obesity populations remains insufficient. Thus, these interventions should currently be considered potentially complementary components of a broader exercise- and nutrition-based strategy rather than a proven synergistic treatment. From a translational perspective, the clinically relevant endpoint should extend beyond changes in body weight to include fat mass, lean mass, muscle strength, physical function, metabolic health, and, where appropriate, neurobehavioral outcomes. This multidimensional approach is consistent with the central framework of this review: exercise provides the primary adaptive stimulus, whereas creatine and EAAs may provide complementary nutritional support for maintaining or enhancing the capacity of skeletal muscle to adapt. Determining which patients derive the greatest benefit from each component—and whether their combination provides incremental benefit—should remain a priority for future clinical trials.

## 10 Limitations of current evidence

Despite increasing interest in combining exercise with creatine and EAA supplementation in overweight and obesity, the current evidence base remains heterogeneous and does not yet establish a standardized combined intervention or a definitive clinical advantage of using creatine and EAAs together. Studies differ substantially in participant characteristics, obesity phenotype, exercise modality and intensity, supplement composition, creatine and amino-acid doses, timing of administration, dietary background, and intervention duration. These differences limit direct comparisons across studies and make it difficult to determine whether observed effects are attributable primarily to exercise, to an individual nutritional component, or to their combination. This issue is particularly relevant because many studies use protein or multi-ingredient formulations rather than isolated EAAs, while relatively few directly compare exercise alone with exercise plus creatine, exercise plus EAAs, and the combined intervention. A second limitation concerns the predominant emphasis on skeletal-muscle outcomes. Most clinical studies assess muscle mass, strength, physical performance, or body composition, whereas the proposed muscle–adipose–brain axis has rarely been investigated as an integrated system. Although myokines, adipokines, exerkines, inflammatory mediators, and circulating metabolites provide plausible routes of communication among skeletal muscle, adipose tissue, immune cells, and the brain, direct evidence that creatine or EAA supplementation modifies these interconnected pathways in humans with obesity remains limited. Consequently, mechanistic links involving adipose remodeling, immune-cell metabolic reprogramming, neuroinflammation, or brain bioenergetics should currently be regarded as emerging or hypothesis-generating rather than established clinical mechanisms.

### 10.1 Population and study-design limitations

Important populations remain underrepresented. Evidence is particularly limited in older adults with obesity, individuals with sarcopenic obesity, adolescents, people with severe obesity, and patients with obesity-associated neuropsychiatric or metabolic comorbidities. Even within sarcopenic obesity, existing intervention studies show substantial methodological heterogeneity, and systematic evidence indicates that the certainty of effects for several outcomes remains limited. Many available trials also include relatively small samples and short intervention periods, which restrict statistical power and provide limited information regarding long-term maintenance, adherence, safety, and clinically meaningful changes in body composition or physical function. Sex-specific responses, age-related anabolic resistance, baseline dietary protein intake, habitual physical activity, metabolic phenotype, medication use, and the coexistence of metabolic syndrome or sarcopenia are also inconsistently accounted for. These factors may contribute substantially to between-person variability and should be incorporated into future trial designs rather than treated simply as sources of unexplained heterogeneity.

### 10.2 Mechanistic and biomarker limitations

The mechanistic evidence linking creatine and EAAs to immunometabolic remodeling of the muscle–adipose–brain axis remains particularly incomplete. Human intervention studies rarely evaluate immune-cell phenotypes, circulating metabolites, mitochondrial bioenergetics, inflammatory signaling networks, or tissue-specific molecular responses within the same experimental framework. Similarly, biomarkers that could provide evidence of inter-organ communication including myokines, adipokines, neurotrophic factors such as BDNF, amino-acid metabolites, and exercise-responsive metabolites, are seldom measured longitudinally alongside clinical outcomes. This limitation is important because associations between circulating biomarkers and clinical outcomes do not necessarily demonstrate causal communication between tissues. For example, changes in circulating metabolites may reflect altered substrate availability, muscle turnover, adipose metabolism, dietary intake, or exercise itself rather than a specific effect of creatine or EAA supplementation. Integrated measurement of circulating metabolites, immune-cell phenotypes, muscle and adipose tissue signaling, and brain-related biomarkers will therefore be necessary to determine whether the proposed muscle–adipose–brain pathways represent clinically relevant mechanisms or primarily conceptual links.

### 10.3 Neurobehavioral and brain-health limitations

Evidence concerning depression, cognition, anxiety, and other neurobehavioral outcomes remains substantially less developed than evidence for muscle and metabolic outcomes. Although obesity is associated with neuroinflammation, altered cerebral metabolism, and increased risk of adverse neurobehavioral outcomes, relatively few trials of creatine or EAA supplementation combined with exercise have incorporated validated measures of depressive symptoms, cognitive performance, emotional regulation, or quality of life. Moreover, direct assessment of cerebral bioenergetics, mitochondrial function, blood–brain barrier integrity, microglial activity, or central insulin signaling is uncommon. Accordingly, the hypothesis that creatine and EAAs enhance exercise-related improvements in brain health through cerebral bioenergetic support, neurotransmitter availability, mitochondrial adaptation, or attenuation of neuroinflammation remains plausible but insufficiently tested. Future trials should therefore treat neurobehavioral outcomes as predefined endpoints rather than extrapolating brain-health benefits from improvements in peripheral muscle or metabolic measures.

### 10.4 Limitations in precision and individualized intervention

Another important limitation is the limited characterization of interindividual variability in response. Potential modifiers include age, sex, baseline muscle mass, habitual protein intake, training status, metabolic phenotype, medication use, dietary pattern, gut microbiota, genetic background, and baseline creatine availability. However, most intervention studies are not adequately powered or designed to identify these response modifiers. Consequently, precision-management approaches involving genotype, microbiome profiles, metabolomics, or other multi-omics measures should currently be regarded as investigational rather than validated tools for selecting creatine or EAA supplementation strategies. Future studies should therefore move beyond simple responder/non-responder classification and prospectively evaluate whether baseline biological characteristics predict clinically meaningful changes in fat mass, lean mass, strength, metabolic health, or neurobehavioral outcomes. Factorial and adequately powered randomized trials will be particularly important for determining whether combined creatine and EAA supplementation provides incremental benefits beyond exercise alone or beyond either nutritional intervention individually. Overall, the major limitation of the current literature is not a lack of plausible mechanisms, but the limited number of well-controlled human studies capable of connecting those mechanisms to clinically meaningful outcomes. Future research should prioritize adequately powered, longer-duration, multicenter randomized trials with standardized definitions of obesity and sarcopenic obesity, clearly characterized exercise protocols, well-defined supplement formulations, and appropriate control groups. Mechanistic substudies incorporating metabolomics, immunophenotyping, body-composition assessment, and, where justified, neuroimaging could then determine whether changes in the muscle–adipose–brain axis mediate clinical responses. Such an approach would help distinguish established effects of exercise and nutritional support from emerging mechanisms and hypothetical pathways, while providing a stronger basis for determining whether creatine and EAAs should be used individually or together in the precision management of obesity.

## 11 Future directions and recommendations

Future research should first establish whether combining creatine and EAA supplementation with structured exercise provides incremental benefits beyond exercise alone or either nutritional intervention individually in individuals with overweight or obesity. Well-designed randomized controlled trials should directly compare exercise alone, exercise plus creatine, exercise plus EAAs, and the combined intervention, preferably using factorial designs that can distinguish independent, additive, and interactive effects. Rather than assuming synergy, these studies should determine whether a reproducible interaction exists and under which conditions it occurs. Standardization of creatine formulation and dose, EAA composition and leucine content, supplementation duration, dietary protein intake, and the timing of supplementation relative to exercise will be important for meaningful cross-study comparisons. Future trials should also prioritize clinically relevant outcomes rather than body weight alone. These should include fat mass, lean mass, muscle strength, physical function, metabolic health, and maintenance of muscle during intentional weight loss. In selected populations, validated measures of depressive symptoms, cognition, quality of life, and other neurobehavioral outcomes should be incorporated as prespecified secondary or exploratory endpoints. Longer-duration studies are particularly important for determining whether improvements in body composition and physical function are maintained after the initial intervention and whether supplementation influences adherence, exercise capacity, or the preservation of muscle during prolonged energy restriction.

A second priority is to determine which obesity phenotypes are most likely to benefit from these interventions. Future studies should prospectively characterize factors such as baseline muscle mass and strength, adiposity distribution, metabolic health, age, sex, training status, habitual protein intake, and baseline creatine availability. Particular attention should be given to populations at increased risk of muscle loss, including individuals with sarcopenic obesity and older adults undergoing weight-loss interventions. Post-bariatric populations, adolescents, and individuals with clinically significant neuropsychiatric comorbidities should be studied cautiously and in appropriately designed trials rather than extrapolated from general obesity cohorts. Mechanistic studies should next determine whether the proposed muscle–adipose–brain axis represents a measurable biological pathway through which exercise and nutritional supplementation influence systemic and neurobehavioral outcomes. Rather than measuring large numbers of biomarkers without a defined mechanistic framework, future investigations should use longitudinal sampling to connect exercise-induced changes in muscle and adipose metabolism with circulating metabolites, myokines, adipokines, exerkines, inflammatory mediators, and clinically relevant outcomes. Stable-isotope tracer approaches, targeted metabolomics, proteomics, and immunophenotyping could help establish whether changes in circulating substrates or signaling molecules reflect altered tissue metabolism and whether they are associated with changes in muscle adaptation or adipose remodeling.

The brain component of this framework also requires direct investigation. Neuroimaging, neurocognitive testing, and, where feasible, measures of cerebral bioenergetics could determine whether peripheral adaptations are accompanied by measurable changes in brain function. Mechanistic hypotheses involving mitochondrial function, cerebral energy metabolism, neurotransmitter availability, BDNF-related signaling, neuroinflammation, microglial activation, blood–brain barrier integrity, and central insulin signaling should be tested directly rather than inferred from peripheral metabolic outcomes. These approaches may clarify whether changes in mood or cognition are mediated primarily by improved physical fitness and metabolic health or whether creatine, EAAs, or their combination exert additional effects on brain physiology. Advanced molecular approaches, including multi-omics integration, transcriptomics, single-cell analyses, epigenomics, microbiome profiling, and spatial approaches, should be applied selectively to address specific mechanistic questions. Such approaches may help identify biological signatures associated with differential responses to exercise and supplementation, but their clinical utility should be established only after reproducible associations have been validated in independent cohorts. Similarly, artificial intelligence, wearable-derived measures, and digital biomarkers may eventually support response prediction and individualized exercise–nutrition prescriptions, but these technologies should initially be treated as research tools rather than validated clinical decision systems. Ultimately, the field should progress from demonstrating efficacy to explaining heterogeneity and then to testing mechanism-informed personalization. Future multicenter trials should therefore integrate standardized exercise protocols and well-characterized nutritional interventions with longitudinal assessments of body composition, muscle function, metabolic health, and, where appropriate, neurobehavioral outcomes. Mechanistic substudies can then determine whether changes in circulating metabolites, immunometabolic signatures, or muscle–adipose–brain communication mediate clinical responses. This stepwise approach will help establish which effects are attributable to exercise, which may be supported by creatine or EAAs, and whether their combination provides clinically meaningful benefits. Until such evidence becomes available, creatine and EAA supplementation should be regarded as potentially complementary nutritional strategies within an exercise-centered approach to obesity management rather than as an established synergistic therapy.

## 12 Conclusion

Current evidence supports exercise as the primary intervention for improving muscle function, body composition, and metabolic health in individuals with overweight or obesity, while creatine and EAA supplementation may provide complementary nutritional support for exercise-induced adaptation. Creatine primarily contributes to cellular energy buffering and may enhance resistance-exercise capacity and training-related gains in strength and lean mass, whereas EAAs provide substrates and nutrient signals that support muscle protein synthesis. Together with structured exercise, these interventions may help preserve or improve functional lean tissue during weight management, particularly when anabolic resistance or energy restriction limits skeletal-muscle adaptation. However, direct evidence demonstrating that creatine and EAAs exert synergistic effects when combined with exercise remains limited, and the relative contribution of each component has not been adequately separated in well-controlled human trials.

Beyond skeletal muscle, exercise-induced myokines, exerkines, adipokines, and circulating metabolites provide a biologically plausible framework for communication among skeletal muscle, adipose tissue, immune cells, and the brain. Preclinical and indirect human evidence suggests that these pathways may be relevant to adipose remodeling, immunometabolic regulation, cerebral bioenergetics, neuroinflammation, cognition, and mood; however, direct evidence linking creatine–EAA supplementation with coordinated changes across the muscle–adipose–brain axis in obesity remains insufficient. Consequently, potential effects on depression, cognitive function, and brain health should be considered emerging research areas rather than established clinical outcomes. The principal research priority is therefore to determine whether combined creatine and EAA supplementation provides clinically meaningful benefits beyond exercise alone or either nutritional intervention individually. Future adequately powered and longer-duration randomized trials should use factorial designs, standardized supplementation protocols, and clinically relevant outcomes encompassing fat mass, lean mass, muscle strength, physical function, metabolic health, and, where appropriate, neurobehavioral measures. Mechanistic studies integrating circulating metabolites, immunophenotyping, multi-omics, and selected neuroimaging approaches may further determine whether changes in muscle–adipose–brain communication mediate these outcomes. At present, creatine and EAAs are best viewed as potentially complementary components of an exercise-centered approach to obesity management, rather than as an established synergistic or precision therapy.
